# A Case of Chorea, Associated with Mitral Insufficiency, Successfully Treated with Digitalis—With Remarks on Chorea

**Published:** 1892-11

**Authors:** Jeff S. Davis

**Affiliations:** Montevallo, Ala.


### A CASE OF CHOREA, ASSOCIATED WITH MITRAL INSUFFICIENCY, SUCCESSFULLY TREATED WITH DIGITALIS—WITH REMARKS ON CHOREA.

BY JEFF S. DAVIS, M. D., MONTEVALLO, ALA.

In April, 1891, Miss M. R. applied to me for treatment for chorea of eight months duration. She had been treated by several physicians, and all the remedies usually administered in this disease had been tried. Among other drugs she had taken Fowler's solution in heroic quantities, having had administered by one physician thirty drops hypodermically, three times daily, for several days.

Antipyrin, the bromides, spinal sprays and electricity had signally failed to relieve or to noticeably mitigate the exhaustive, incessant, clonic spasms, which as she expressed it, were gradually "shaking her life away." The following history of the case, copied from my case book, was furnished by her mother, who was of marked nervous temperament:

Age 19; father died of phthisis pulmonalis, age 51; patient very anæmic since one year of age; had a protracted case of typhoid fever when ten years old, which was followed by a cough of eighteen months duration; menstruated at fifteen, but was never regular, a period of from three to four months usually intervening the catamenial epochs, which were always menorrhagic in character; had suffered repeated attacks of acute articular rheumatism during the preceding two years—the last nine months prior to the time that she came under my care; had been coughing several weeks and expectorating sputa tinged with blood. Immediately after the subsidence of the last attack of rheumatism the first choreic symptoms were noticed. The facial muscles were first affected, and very soon afterwards those of the neck and extremities of both sides. The contractions, which were at first slight, gradually became more pronounced until locomotion was impossible. On examination I found anasarca of lower limbs, dyspnœa, pain in precordial region, constipation, tongue large and pale, pressure 140 and very weak, respiration hurried. Physical examination revealed bilateral mucous rales and a mitral systolic murmur. The murmur was unusually loud and propagated towards the left axilla, and quite audible in the left vertebral groove. Mitral insufficiency was painly evident, and having determined the cardiac lesion, the anasarca, bronchitis and dyspnœa were recognized at once as patent symptoms of that affection. The chorea, however, could not be so readily assigned a course, and the whole category of recognized remedies having been exhausted without materially effecting its course, the prognosis was naturally unfavorable. Realizing the importance of a plan of treatment essentially different from any adapted by my predecessors, I studied particularly the etiological aspect of the case for any knowledge that would suggest a definite line of treatment. The conclusion finally arrived at was, that malnutrition of long standing intensified by the valvular trouble were the principal etiological factors in the case. The following was accordingly directed:

R. Tr. digitalis,................................oz. ii
Tr. ferri chlor.,................................oz. i
Glycerine,.......................................oz. iii
M. Sig. One teaspoonful. t. i. d.

The efficacy of this combination can be seen at once. The digitalis restoring the relation between arteries and veins through its specific action on the heart, thereby increasing the quantity of oxygen and blood distributed to the various tissues, while the iron, by building up the histological elements of the blood, tends to obviate the results arising from a diminished quantity due to the cardiac lesion. A highly nutritive, easily digested diet was directed. Ordered her to take food in sufficient quantities to appease appetite, and in addition to this to take each day three soft boiled eggs and one pint of fresh cream, as blood material. The two latter were to be taken over and above the quantity called for by her appetite. For the constipation and to promote digestion, prescribed:

R. F. E. cascara sag.,.............................dr. iv
Elix. lactopep.,...................................oz. vii ss
M. Sig. Tablespoonful after meals.

For insomnia, she was given sulfonal, gr. xviii, at bedtime. The glandular system received scrupulous attention, small doses of calomel being frequently given when indicated.

A detailed account of the daily progress of the case is not necessary. Suffice to say, that the fifth day after beginning the treatment there was marked improvement. Dyspepsia very slight, cough less frequent and sputa only sparingly stained; pulse 98 with good volume; anasarca disappearing and a decided abatement in the involuntary muscular contractions. With the exception of discontinuing the sulfonal, no change was made in the treatment at this time. Improvement was uninterrupted, and by the twentieth day there was no symptom of chorea, no bronchitis, no anasarca. A great change had been effected in her appearance in this short time.

The eye, which had regained a degree of its wonted brilliancy; the wan cheek delicately tinted by the rosy hue of health, together with the increased color in the pallid lips, bore evidence that nutrition was much improved. As the kidneys were functionally active, I apprehended no danger from the reputed accumulative effect of the digitalis, and when my visits were discontinued, after the thirtieth day, she was directed to continue the treatment and requested to report at my office every two weeks. As too often happens in such cases, where directions are not enforced by rigid supervision, she was soon lulled into a state of indifference by the erroneous idea that she was cured, and all treatment abandoned. With a mitral lesion in a non-compensating heart, the result of withdrawing the digitalis may be easily imagined. Re-establishment of the previous symptoms rapidly ensued, and the chorea was, if possible, more severe than when I first saw her The same course of treatment detailed above was directed, and while the case did not yield so readily to treatment as in the first instance, her condition was in two months as favorable as before the relapse.

Having profited by this experience, she realized the importance of continued treatment, and no further trouble was experienced in having her follow directions.

When the defecatory functions were normally established, the cascara sagrada was discontinued, and cod-liver oil given before meals with elixir lactopep., dr. iv, immediately after meals. Before coming under my care she had been treated for rheumatism, and there has been no symptom of that disease for more than a year. She has taken the digitalis and iron mixture and lactopeptine almost continuously since I first saw her. Cod-liver oil is given until it begins to nauseate, when it is left off, and the hypophosphites given for two or three weeks.

Her condition is at present almost incredulously good, and notwithstanding the fact that she has a grave cardiac affection, is able to engage temperately in the various pleasures of life. The chorea is apparently permanently cured, though in my opinion a relaxation in treatment would be speedily followed by its return.

REMARKS—The history of chorea dates back to the middle ages, and from that time to the present day many theories have been formulated to explain its cause, and many methods advocated for its abolition. To transcribe the various conditions considered as having an etiological relation to chorea is unnecessary, as the literature on the subject is voluminous and accessible to the student. Pathologically considered, the disease is apparently on the border line between the functional and the organic; post mortem investigations revealing in some cases anotomical characters, while in other cases no such characters are discoverable.

"Dr. Dickson, in a large series of examinations, found dilatation of minute arteries existing throughout the brain and cord, more especially in the corpus striatum and thalamus with small hemorrhage, and believed the disease to be due to a widespread hyperæmia of the nerve centres." (Broadbent).

The embolic theory of King and Jackson is familiar to all, while rheumatism and its sequels are so intimately associated with chorea that more able investigators have considered the disease essentially rheumatic.

From the fact that in "almost all fatal cases of chorea there exists endocarditis with deposits of beads of lymph on the mitral or aortic valves," the cardiac and embolic connection and its relation with rheumatism is readily appreciable. Yet there are other cases preceding or co-existing with an attack of rheumatism where there is no evidence of endocarditis, which are obviously due to causes other than the lodgment in a cerebral vessel of fibrinous shreds from an inflamed endocardium. In many cases nothing abnormal has been detected after death. Pregnancy, intestinal worms, onanism, and even anæmia in one predisposed, are often the only conditions present to which the disease may be attributed. It occurs more frequently in families in which nervous diseases are hereditary; and bad hygienic surroundings, together with improper food play no unimportant part in its production. Of the remedies for chorea, the preparations of arsenic are the most potent, and the majority of cases will be cured when they are properly administered. That it is not always amenable to this treatment, however, is evinced by the case reported. But the scope of this paper will not permit an elaborate discussion of the etiology and treatment of chorea, and the above remarks are only intended to remind the reader that no one condition or agent can be accepted as the only cause of chorea, and consequently no single line of treatment expected to prove infallible. Where possible, remedies should be administered to meet the indications in each individual case, in this disease as in all others. The fact that the "irregular clonic muscular spasms" is a morbid manifestation or symptom, and not a disease, *per se*, should be kept constantly in mind.

A desire to call attention to the benefit derived from digitalis in the treatment of chorea associated with valvular lesions, is the motive which prompted the report of the above case. That such cases are of rare occurrence, I admit, but when the conditions attending mitral insufficiency are taken into consideration, its importance as an etiological factor in chorea is at once apparent. That anæmia is a recognized accompaniment of chorea, and in some cases the only discoverable cause, all will admit, and it is reasonable to suppose that mitral insufficiency, or rather the anæmia resulting from the diminution of oxygenated blood in the tissues, may in itself or by intensifying pre-existing anæmia, determine an attack of chorea in one predisposed. In such cases, every effort should be made to improve *nutrition*, and in addition to other treatment, digitalis by restoring the relation between arteries and veins, will prove very advantageous. It will effect cures when all else has failed. It should be given in *every* case of chorea associated with a heart lesion for which the drug would be ordinarily prescribed, and in these cases it is as distinctively curative in its effects as Fowler's solution.

